# Modulation of Neuroimmune and Epithelial Dysregulation in Patients With Moderate to Severe Prurigo Nodularis Treated With Nemolizumab

**DOI:** 10.1001/jamadermatol.2023.2609

**Published:** 2023-08-09

**Authors:** Junwen Deng, Viviane Liao, Varsha Parthasarathy, Hannah L. Cornman, Anusha Kambala, Madan M. Kwatra, Sonja Ständer, Christophe Piketty, Prasad Chaskar, Jayendra Kumar Krishnaswamy, Valerie Julia, Shawn G. Kwatra

**Affiliations:** 1 Department of Dermatology, Johns Hopkins University School of Medicine, Baltimore, Maryland; 2 Department of Anesthesiology, Duke University School of Medicine, Durham, North Carolina; 3 Department of Dermatology and Center for Chronic Pruritus, University Hospital Münster, Münster, Germany; 4 Galderma SA, Lausanne, Switzerland

## Abstract

**Importance:**

Prurigo nodularis (PN) is a debilitating skin disease characterized by intense pruritus and hyperkeratotic skin nodules. Nemolizumab, a monoclonal antibody targeting interleukin 31 receptor α, is a promising novel therapy for the treatment of moderate to severe PN. The biological mechanisms by which nemolizumab promotes improvement of itch and skin lesions in PN are unknown.

**Objective:**

To characterize changes in plasma protein biomarkers associated with clinical response to nemolizumab in patients with PN.

**Design, Setting, and Participants:**

This multicenter cohort study included patients recruited from Austria, France, Germany, Poland, and the US from a phase 2 clinical trial. Adults diagnosed with moderate to severe PN with severe pruritus for at least 6 months were included in the original trial. Patients in the nemolizumab group were included in the present study if they achieved at least a 4-point decrease in the Peak Pruritus Numerical Rating Scale (PP-NRS) from baseline to week 12 during nemolizumab treatment. Placebo controls did not experience a 4-point decrease in PP-NRS. Mass spectrometry with tandem mass tags to enrich skin-specific protein detection was used to characterize changes in plasma protein expression in nemolizumab and placebo groups. Data were collected from November 2, 2017, to September 26, 2018, and analyzed from December 6, 2019, to April 8, 2022.

**Intervention:**

As part of the clinical trial, patients were treated with 3 doses of nemolizumab or placebo at 0, 4, and 8 weeks.

**Main Outcomes and Measures:**

Changes in plasma and epidermal protein expression in nemolizumab-treated patients compared with the placebo group at 0, 4, and 12 weeks.

**Results:**

Among the 38 patients included in the analysis (22 women and 16 men; mean [SD] age, 55.8 [15.8] years), enrichment analysis of canonical pathways, biological functions, and upstream regulators showed downregulation of terms involving inflammation (IL-6, acute-phase response, signal transducer and activator of transcription 3, and interferon γ), neural processes (synaptogenesis signaling and neuritogenesis), tissue remodeling and fibrosis (transforming growth factor β1 and endothelin-1), and epidermal differentiation (epithelial mesenchymal transition) in the plasma of nemolizumab group.

**Conclusions and Relevance:**

In this cohort study, differences between nemolizumab and placebo groups included modulation of inflammatory signaling, neural development, and epithelial differentiation, suggesting a promising potential approach for clinical management of PN.

## Key Points

**Question:**

What is the association of interleukin 31 receptor α (IL-31RA) inhibition with plasma protein expression in nemolizumab treatment of prurigo nodularis (PN)?

**Findings:**

In this cohort study of 38 patients, plasma samples were collected from the nemolizumab response and placebo nonresponse groups from a phase 2 clinical trial of PN. Analysis of 193 differentially expressed plasma proteins demonstrated an association of nemolizumab with epidermal differentiation, tissue remodeling and/or fibrosis, neural dysregulation, and inflammation.

**Meaning:**

These findings suggest that nemolizumab response is associated with modulation of inflammatory, neural, and epithelial signaling compared with placebo nonresponse, demonstrating the multifaceted role of IL-31RA inhibition in the treatment of PN.

## Teaser

This cohort study analyzes the underlying changes in protein biomarkers and pathways in plasma samples associated with clinical improvement due to nemolizumab treatment among patients with prurigo nodularis.

## Introduction

Prurigo nodularis (PN) is a debilitating chronic inflammatory skin disease characterized by chronic pruritus and the presence of multiple localized or generalized pruritic, hyperkeratotic nodules that favor the trunk and extremities.^1,2^ Patients with PN often experience a negative effect on their quality of life due to the intense and chronic itch, pain, and loss of sleep associated with the disease.^3,4,5,6^ While the exact pathogenesis of PN remains unclear, immune and neural dysregulation play significant roles.^7,8^ Immunophenotyping studies of PN^9,10,11,12^ show increased T lymphocytes, mast cells, and eosinophils associated with PN, as well as increased inflammatory cytokines including interleukin 4 (IL-4), IL-13, IL-31, IL-17, IL-22, and IL-31. Prurigo nodularis is also associated with disturbed neuronal architecture (increased epidermal neuronal branching and dermal neuronal hyperplasia) and dysregulation of neuropeptides that may contribute to inflammation.^13,14,15,16^ Epidermal dysregulation is seen as a result of these processes, characterized by epidermal hyperplasia and hyperkeratosis, as well as proinflammatory vascularization processes in the dermis through activation of vascular endothelial growth factor (VEGF).^17,18,19^

Targeting the IL-31 receptor α (IL-31RA) in PN with subcutaneous injection of nemolizumab, 0.5 mg/kg every 4 weeks, improved clinical responses in adults with moderate to severe PN in a randomized, double-blind phase 2 clinical trial.^20^ Interleukin 31 is a neuroimmune cytokine that is associated with sensory neuronal outgrowth and stimulation, inflammation, and altered epidermal differentiation. The receptor consists of the IL-31RA and the oncostatin M receptor β chain. Binding of IL-31 to its heterodimeric receptor leads to activation of the Janus kinase–signal transducer and activator of transcription (STAT) pathways that affect inflammation, pruritus, epidermal differentiation, and fibrosis.^9^ In addition to dorsal root ganglions, IL-31RA is expressed by a variety of inflammatory and structural cells, including myeloid cells, granulocytes, and keratinocytes.^21^ A recent immunofluorescence analysis revealed that most IL-31RA–expressing dermal cells in PN lesions were mast cells and macrophages.^22^

In the phase 2 trial for nemolizumab, patients with moderate to severe PN received either subcutaneous injections of the active compound at a dose of 0.5 mg/kg of body weight or placebo at baseline, week 4, and week 8. In the trial, the nemolizumab group demonstrated significant improvements in the Peak Pruritus Numerical Rating Scale (PP-NRS) score, number of lesions, investigator’s global assessment (IGA), sleep quality, and dermatology life quality index.^20^ Given the significant clinical efficacy of nemolizumab, this study aims to analyze the underlying changes in protein biomarkers and pathways in plasma samples associated with clinical improvement due to nemolizumab treatment.

## Methods

### Study Design and Oversight

This cohort study was conducted on a subset of patients who achieved clinical improvement from the multicenter, placebo-controlled, double-blind phase 2 randomized clinical trial involving patients with moderate to severe PN.^20^ The trial was conducted according to the principles of the Declaration of Helsinki,^23^ and the protocol was approved by ethics committees at each participating institution, and patients provided written informed consent. This study followed the Strengthening the Reporting of Observational Studies in Epidemiology (STROBE) reporting guideline.

### Patient Population

Patients were recruited from centers in Austria, France, Germany, Poland, and the US. Inclusion criteria for patients in the clinical trial included adults with severe pruritus, defined as a mean score of at least 7 over the previous week on the PP-NRS (ranging from 0 [no itch] to 10 [worst itch imaginable]), for at least 6 months. Patients were required to have moderate to severe PN, which was defined as nodular lesions on the upper extremities, with or without lesions on the trunk or lower extremities, at least 20 nodules on the body, and a distribution of lesions present on both sides of the body. Patients were excluded if they had chronic pruritus due to any condition other than PN, unilateral lesions, or signs of neuropathic or psychogenic pruritus.

Data were collected from November 2, 2017, to September 26, 2018. Patients from this trial were included in the present study as having a nemolizumab response if they achieved at least a 4-point decrease (improvement) of PP-NRS score from baseline to week 12 while receiving nemolizumab. Patients were included as having a placebo nonresponse if they were in the placebo arm of the clinical trial and did not experience at least a 4-point decrease in PP-NRS score. Furthermore, IGAs for patients at week 18 were also described, where a successful response was defined as a score of 0 or 1 plus an improvement of 2 points from baseline. Detailed patient characteristics, reflecting no significant differences in demographics or baseline disease severity, are shown in the Table.

**Table.** Patient Demographic and Clinical Characteristics

Characteristic	Treatment group^a^		*P* value^b^
	Placebo nonresponder (n = 19)	Nemolizumab responder (n = 19)	
Sex			
Men	7	9	.51
Women	12	10	
Race			
Black or African American	1	1	>.99
White	18	18	
Mean (SD) age, y	55.6 (18.3)	59.2 (12.9)	.48
Baseline PP-NRS, mean (SD)^c^	8.45 (0.10)	8.33 (1.10)	.72
Sleep disturbance NRS, mean (SD)^c^	6.96 (2.39)	6.87 (1.54)	.89
IGA (ordinal logistic)			
Moderate	12	11	.74
Severe	7	8	

Abbreviations: IGA, investigator’s global assessment; PP-NRS, Peak Pruritus Numerical Rating Scale.

^a^ Unless otherwise indicated, data are expressed as No. of patients.

^b^ Calculated as χ^2^ test for sex and race.

^c^ Scores ranged from 0 (no itch) to 10 (worst itch imaginable).

Plasma samples from patients with PN were collected at baseline, week 4, and week 12 from participants receiving the active compound (n = 19) or placebo (n = 19). Skin biopsy specimens from 4 healthy individuals were also provided as trigger material for tissue-related protein analysis. Skin samples were stored at −80°C until analysis. Plasma samples and trigger material were used for protein mass spectrometry followed by differential expression analysis and enrichment analysis, as outlined in eFigure 1 in Supplement 1.

### Proteomics Methods: Protein Mass Spectrometry

Samples were depleted of highly abundant proteins using high-performance liquid chromatography–assisted human IgY14 and SuperMix (Scientific Software International) columns to remove approximately 65 high- to medium-abundant plasma proteins. Each depleted plasma sample (25 μg) was digested and labeled with 1 of the first 12 proline-based reporter isobaric tandem mass tag (TMTpro) reagents. In parallel, aliquots of a skin biopsy lysate were digested and labeled with the remaining TMTpro reagents (25, 100, 150, and 250 μg) and mixed with the analytical samples to form a TMTpro 16plex for a total of 10 TMTpro 16plexes. Each plex consisted of equal amounts equivalent to 1 proteome unit for the 12 depleted plasma samples (analytical channels) and 1, 4, 6, and 10 proteome units for the 4 tissue samples from healthy individuals (calibrator and/or trigger channels). The 10 mixed plexes were separated using basic reverse-phase chromatography into 30 fractions per plex. Each fraction was subjected to liquid chromatography–mass spectrometry or mass spectrometry analysis using a high-performance mass spectrometer (Orbitrap Fusion Tribrid; ThermoFisher Scientific). Raw files were searched within the unifying protein informatics platform (Proteome Discoverer; ThermoFisher Scientific). To improve the quality of comparisons, a baseline subtraction was performed for each patient. Data were further processed using a proprietary bioinformatics pipeline involving filtering, normalization, statistical testing, annotation, and functional analysis. Only peptides that corresponded to a unique protein were used for the statistical analyses.

### Statistical Analysis

#### Exploratory Analysis

Data were analyzed from December 6, 2019, to April 8, 2022. For the exploratory analysis of protein data, we visualized sample-to-sample distance metrics and the results of principal component analysis (PCA). Baseline-correction of data at weeks 4 and 12 was performed by dividing the posttreatment abundance values by the baseline abundance. Sample-to-sample Euclidean distance metrics were computed using the dist() function from the stats R package with default parameters (R, version 3.5.2; R Project for Statistical Computing). Protein abundance values in the heat maps were mean-centered, scaled, and ordered by hierarchical clustering using the sample-to-sample Euclidian distances. Principal component analysis was performed on normalized abundance values using prcomp() function from stats R package with default parameters. For visualization of PCA, we used the ggplot2 and ggfortify R packages.

#### Differential Expression Analysis

Differential expression analysis of protein data used LIMMA-based linear models (using the LIMMA R package) with the following fixed factors incorporated into the models to account for patient variability: class (treatment and time point), batch, sex, and age. Differentially expressed features were identified through a moderated *t* test. Least squares, robust regression, and generalized least squares fitting methods were implemented in LIMMA. Adjusted *P* value and log_2_ fold change were assigned based on the most significant test. For all analyses, an adjusted 2-sided *P* = .05 (Benjamini-Hochberg procedure) cutoff was used to select differential features.

#### Enrichment Analysis

We found 193 plasma proteins were differentially expressed at week 4 or week 12 in patients treated with nemolizumab vs placebo (adjusted *P* < .05). Enrichment analysis was performed using the 193 proteins found differentially expressed with the use of Ingenuity Pathway Analysis (QIAGEN) to discover biological processes (ontology terms) regulated by the proteins of interest. We used the following ontology maps for identification of functional metabolic and signaling processes: Metabase Pathway Maps, Metabase Pathway Maps Folders, and Metabase Process Networks.

## Results

### Clinical Efficacy of Nemolizumab

**Figure 1.**
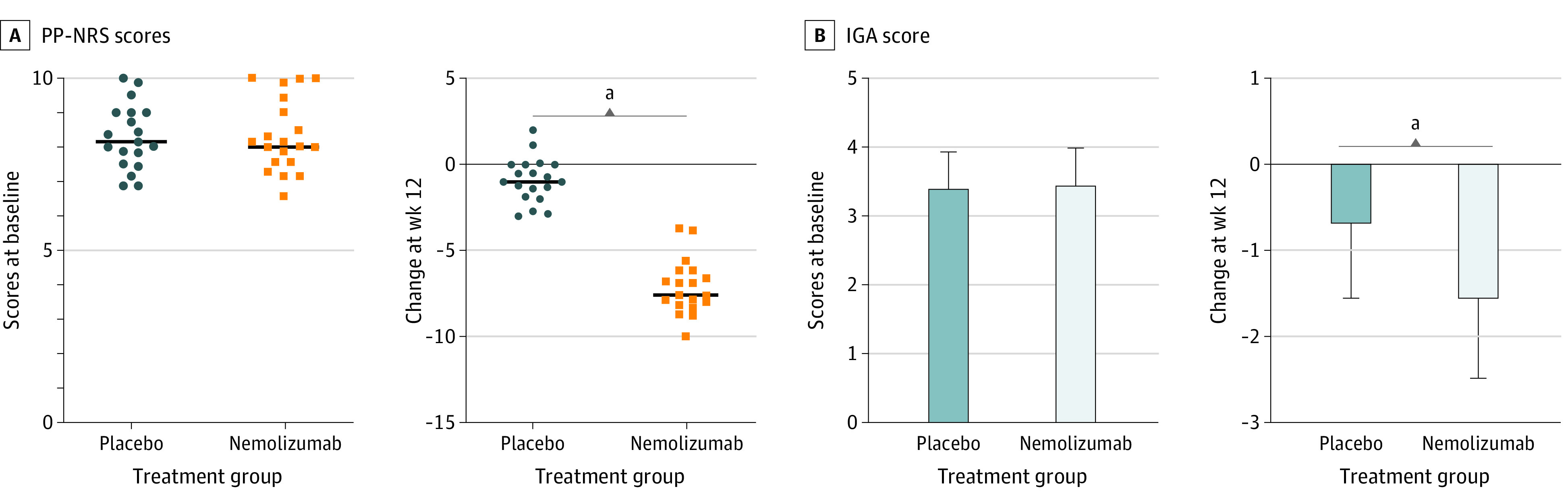
Clinical Efficacy of Nemolizumab A, Baseline Peak Pruritus Numerical Rating Scale (PP-NRS) scores and percentage of patients with no response to placebo and those with a response to nemolizumab treatment at week 4. A successful response according to the PP-NRS was defined as a decrease (improvement) of 4 points from baseline. Horizontal lines indicate mean. B, Baseline investigator global assessment (IGA) scores and percentage of placebo and nemolizumab group patients with IGA success at week 18. A successful response according to the IGA was defined as a score of 0 or 1 plus a decrease (improvement) of 2 points from baseline. Error bars indicate SD. ^a^*P* < .001.

This cohort study is an analysis of a subset of 19 patients treated with placebo and 19 patients treated with nemolizumab from the randomized double-blind phase 2 clinical trial of nemolizumab in moderate to severe PN.^20^ Of these 38 patients, 16 were men and 22 were women; the mean (SD) age was 55.8 (15.8) years. Two patients self-reported as Black and 36 as White. Baseline PP-NRS and IGA scores for both placebo and nemolizumab groups confirm that there were no significant differences at baseline. Within the subgroups selected, 17 of 19 patients in the nemolizumab group (89%) experienced at least 4 points decrease in PP-NRS compared with 0 of 19 in the placebo group at week 4 (*P* < .001) (Figure 1A). Similarly, the frequency of IGA success at week 18 was higher in the nemolizumab group (10 of 19 [53%]) compared with the placebo group (0 of 15) (*P* < .001) (Figure 1B). These results are in line with the previously published study on the whole cohort,^20^ demonstrating that the subgroup selected is representative of the larger cohort.

### Proteomic Analysis of Patients Treated With Nemolizumab

**Figure 2.**
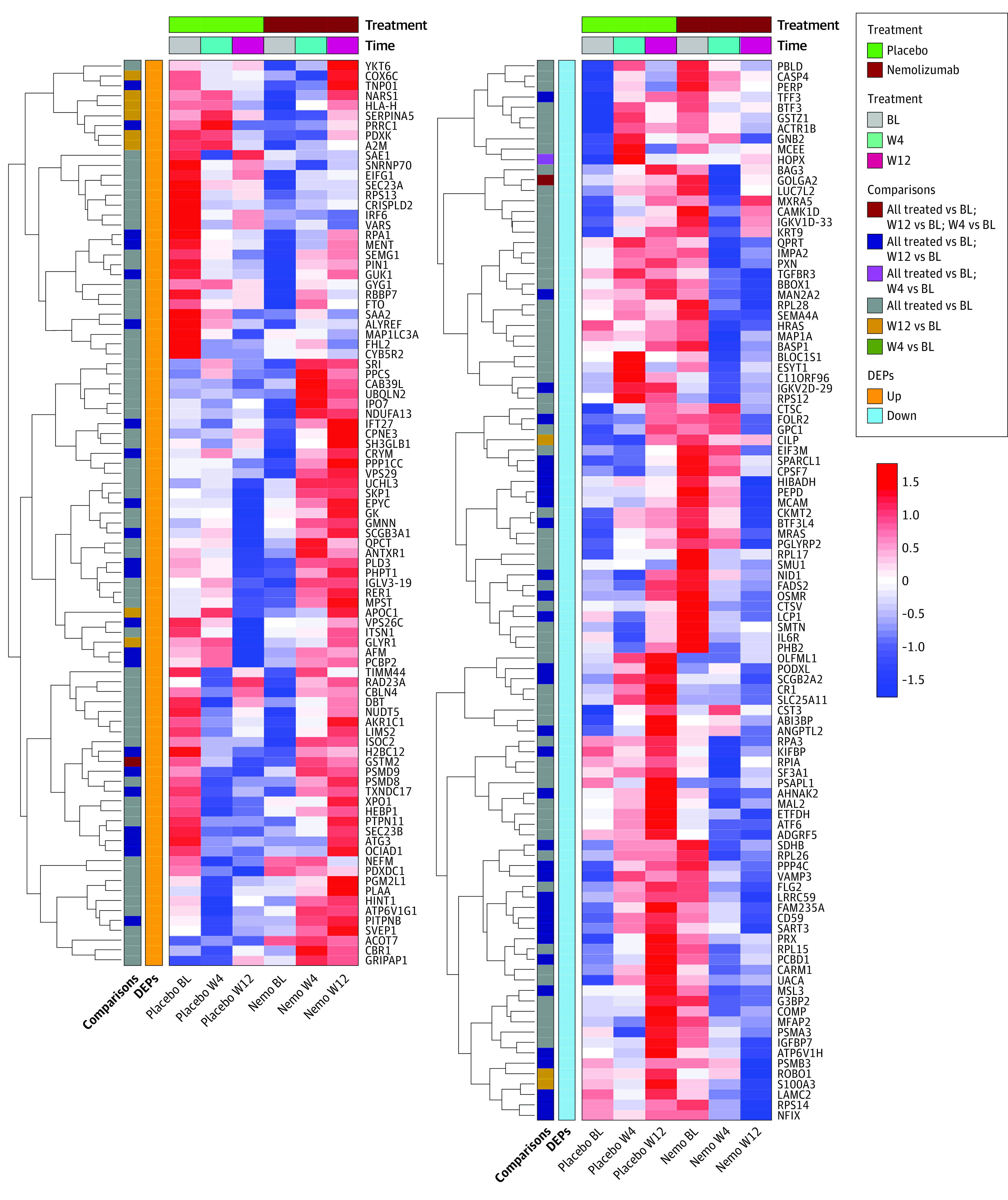
Exploratory Analysis of Differentially Expressed Proteins in the Pooled Nemolizumab (Nemo) Group vs the Placebo Group Heat map of mean abundances for the 193 upregulated and downregulated proteins for each time point (baseline [BL], week 4 [W4], and week 12 [W12]) and treatment combination. Protein abundance values shown in the heat map are mean-centered, scaled, and ordered by hierarchical clustering using Euclidian distances. Red colors represent higher-abundance values while blue colors represent lower-abundance values. All treated terms refer to all treatment time points (ie, week 4 and week 12) being jointly considered for the modeling, as opposed to separately considering week 4 and week 12 for the analysis. DEPs indicates differentially expressed proteins.

**Figure 3.**
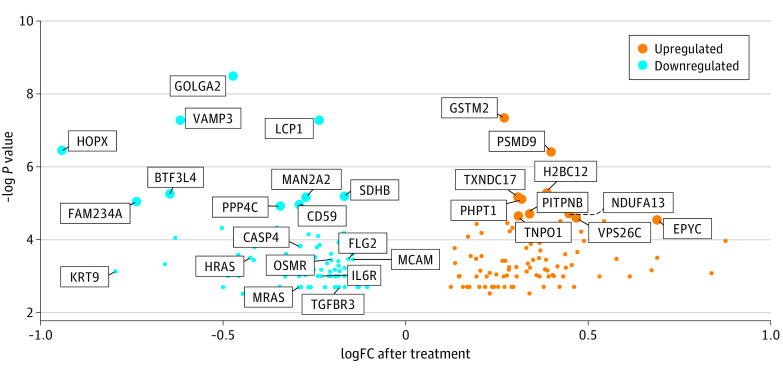
Volcano Plot Representing Selected Upregulated and Downregulated Baseline-Corrected Differentially Expressed Proteins in the Nemolizumab Group vs the Placebo Group LogFC indicates log_2_ fold change.

A total of 193 proteins were differentially expressed after nemolizumab treatment compared with placebo (eTable in Supplement 1). Heat maps of the differentially upregulated and downregulated proteins (eFigure 2A and 2B in Supplement 1) demonstrate distinct trends in protein expression between each of the individuals in the nemolizumab and placebo groups after 12 weeks of treatment. The PCA plots for the normalized abundance values demonstrate that 26.28% of the variance can be explained by the first 2 principal components (eFigure 3A and 3B in Supplement 1). The samples were pooled to visualize overall trends in protein expression in both treatment groups across all different time points and treatment combinations (Figure 2). Figure 3 marks the 10 most upregulated and 10 most downregulated proteins in patients treated with nemolizumab measured at week 4 or week 12 vs baseline, as well as selected differentially expressed proteins involved in inflammatory activation (CD59, caspase 4,and oncostatin M), cellular growth and differentiation (transforming growth factor β [TGF-β] receptor, H-Ras, and M-Ras), or tissue structure and remodeling (fillagrin 2, keratin 9, and melanoma cell adhesion molecule). The aggregate data were then used for enrichment analysis of canonical pathways, biological functions, and upstream regulators.

**Figure 4.**
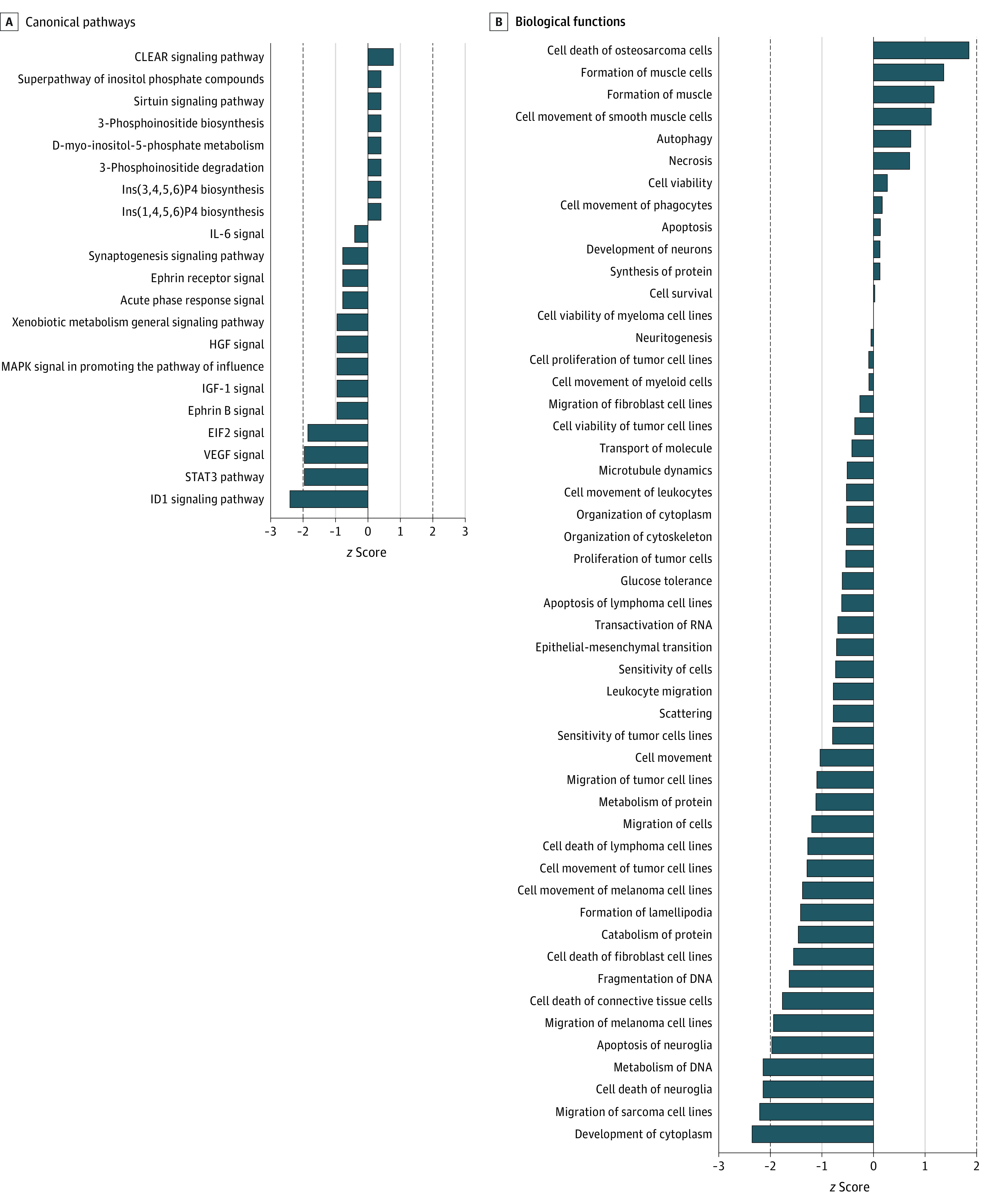
Enrichment Analysis of the Differentially Expressed Proteins Using Ingenuity Pathway Analysis Data were sorted by *z* scores (*z* scores ±1) and filtered by adjusted *P* < .05.

Analysis of canonical pathways in the nemolizumab vs placebo groups, sorted by *z* score and filtered by adjusted *P* < .05, revealed downregulation in inflammatory signaling and pruritus and/or neural dysregulation in the nemolizumab group (Figure 4A). With regard to inflammatory signaling, IL-6 (*z* score = −0.447), acute phase response (*z* score = −0.816), VEGF (*z* score = −2.000), and STAT3 (*z* score = −2.000) pathways were downregulated. Nemolizumab treatment was also associated with increase in terms such as *regulation of the epithelial mesenchymal transition by growth factors*, suggesting an improvement in altered epidermal differentiation in PN—a facet observed in a previous transcriptomic analysis of PN skin by Tsoi et al.^24^ Finally, *synaptogenesis signaling pathway* (*z* score = −0.816), a neuronal ontology, was downregulated in the nemolizumab group, emphasizing the importance of IL-31 as a neuroinflammatory cytokine in PN.

Biological function analysis of the nemolizumab group compared with the placebo group showed similar results (Figure 4B), with downregulation in inflammatory signaling, pruritus and/or neural dysregulation, and epithelial differentiation pathways. With respect to inflammatory signaling, the terms *myeloid cell movement* (*z* score = −0.111), *cell movement of leukocytes* (*z* score = −0.538), and *leukocyte migration* (*z* score = −0.799) were decreased in nemolizumab responders, suggesting that nemolizumab treatment affected cellular infiltration. With regard to neural pathways, there was a notable decrease in cell death of neuroglia (*z* score = −2.177), with slight downregulation of terms such as *neuritogenesis* (*z* score = −0.069) and upregulation of *neuron development* (*z* score = 0.166). Finally, terms related to epithelial differentiation and fibrosis, such as *epithelial-mesenchymal transition* (*z* score = −0.73), *cell death of fibroblast cell lines*, (*z* score = −1.565), and *cell death of connective tissue cells* (*z* score = −1.789), were downregulated. These data suggest that nemolizumab treatment affects various processes involved in PN pathophysiology and that by using mass spectrometry proteomics, we can capture skin-specific signatures in the plasma.

Finally, upstream regulators were analyzed in the nemolizumab group compared with the placebo group to understand how potential transcription factors and other important small molecules may be affected (eFigure 4 in Supplement 1). Major inflammatory mediators affected include interferon γ (*z* score = −0.108), TGF-β1 (*z* score = −1.188), STAT5b (*z* score = −1.633), and endothelin-1 (*z* score = −2.183), which were all downregulated. Endothelial growth factor receptor, involved in tissue remodeling, was upregulated (*z* score = 1.276).

## Discussion

In this cohort study, proteomic analysis of plasma from patients with PN responsive to nemolizumab identified 193 proteins that were differentially expressed after treatment compared with the placebo group of nonresponders, implicating downregulation of inflammatory pathways, neural processes, tissue remodeling and fibrosis, and epidermal differentiation. Among canonical signaling pathways analyzed, downregulation of IL-6 signaling and STAT3, a signal transducer downstream of IL-31, confirms IL-31RA was successfully targeted. Downregulation of STAT5b signaling further reinforces the notion that pathways downstream of IL-31 signaling were affected.^25^ Additionally, VEGF signaling, a driver of vascular proliferation that correlates with PN severity and inflammation,^17^ was downregulated. Endothelin-1, a precursor to the pruritogen endothelin-1 that is upregulated in the epidermis of patients with PN, was also downregulated.^26^ This suggests normalization of endothelin-1 following treatment and may also indicate a connection between the helper T cell 17 (T_H_17) axis and IL-31, as endothelin-1 expression is induced by IL-17 in PN. Likewise, IL-31 has been correlated with T_H_17 cytokines in psoriatic arthritis,^27,28^ and nemolizumab has been shown to effectively decrease T_H_17 and IL-17 responses.^24^ Downregulation in acute phase response signaling, myeloid cell movement, leukocyte migration, and the proinflammatory cytokine, interferon γ, further support a decrease in systemic inflammation.^29,30^ Altogether, these results show plasma biomarkers of inflammation were downregulated in the nemolizumab group compared with the placebo group.

With respect to neural pathways, synaptogenesis signaling and neuritogenesis were significantly downregulated after treatment with nemolizumab. The pathogenesis of PN involves intraepidermal nerve fiber hypoplasia with increased branching of nerve fibers and increased dermal levels of neuropeptide signaling, via factors such as substance P and nerve growth factor.^15,16,31^ Interleukin 31 can activate transient receptor potential vanilloid 1–positive nociceptive neurons of the peripheral nervous system, which leads to transmission of pruritus sensation and enhances inflammation.^32,33^ Decrease in neuronal synaptogenesis pathways following treatment with IL-31 inhibition possibly reflects a decrease in the elevated dermal neuropeptide signaling that is characteristic of PN and restoration of normal neuron density.^7,34^ Furthermore, the decrease in neuroglia cell death after nemolizumab treatment supports a recovery from pathologic intraepidermal nerve fiber hypoplasia and also reflects wound healing, as peripheral glial cells promote wound contraction and healing as mediated by TGF-β.^35^

Pathways related to wound healing and epithelial differentiation also suggest a decrease in epidermal differentiation and fibrosis. Endothelial growth factor receptor, which stimulates epidermal regeneration and is vital to wound healing, was upregulated.^36^ Transforming growth factor β, which is activated and upregulated in fibrotic diseases, was also downregulated,^37,38,39^ which is concordant with the decrease in fibrotic lesions associated with nemolizumab treatment.^20^ This may also be related to a decrease in inflammation, as TGF-β is important in activation of inflammation through T_H_17 cells as well as IL-9–producing T cells.^40,41,42^ Additionally, pathways involved in epidermal differentiation were downregulated in the nemolizumab group, including death of connective tissue cells and epithelial mesenchymal transition. Patients with PN at baseline have previously been found to show an upregulation in epithelial mesenchymal transition, especially compared with patients with psoriasis or atopic dermatitis.^18^ Epithelial mesenchymal transitioning, a process commonly elevated in inflammation and mediated by TGF-β, leads to tissue fibrosis if overactivated and may reflect a decrease in dermal fibrosis among the nemolizumab group compared with the placebo group.^43^

Importantly, this proteomics study demonstrates that systemically, nemolizumab response was associated with improved clinical severity of PN through mediation of acute-phase responses and tissue remodeling, neural development, and epidermal differentiation, compared with placebo nonresponse. These effects are in addition to previous studies of the skin of patients with PN treated with nemolizumab,^24^ which showed IL-31RA inhibition is associated with a downregulation of cutaneous T_H_2 and/or IL-13 responses.

### Limitations

A limitation of our study is that it was performed on a subset of 19 patients in a nemolizumab response group and 19 in a placebo nonresponse group from the original phase 2 clinical trial for PN. Patients who did not respond to nemolizumab treatment and placebo-treated patients with a treatment response were excluded in this exploratory study to better identify the most important and distinct biomarkers associated with clinical response to nemolizumab treatment. This design choice, however, eliminates the benefits of randomization within the trial and opens the possibility for a portion of the observed effects to be derived from intrinsic baseline differences between the 2 groups. Given recent proteomics studies identifying inflammatory and neuropathic subsets of PN,^44^ an important baseline difference limiting generalizability could be differing proportions of PN subsets between the 2 groups and compared with the original patient cohort. This clinical trial, and nearly all others involving PN, however, excluded patients with a potential neuropathic etiology of pruritus, limiting the amount of disease heterogeneity in the original cohort. Furthermore, we have taken steps to mitigate the limitation posed by our group design by ensuring the patient groups had similar baseline clinical characteristics (ie, IGA and PP-NRS scores) to each other and the original patient cohort and by applying stringent measures for our statistical models to take into consideration batch, age, sex, and class (treatment and time point combination). Investigations including larger, fully randomized patient cohorts and further functional studies would be required, however, to determine whether these conclusions can be definitively applied to nemolizumab response in patients with PN.

## Conclusions

In this cohort study, analysis of 193 differentially expressed proteins in the nemolizumab response group compared with the placebo nonresponse group showed changes in signaling pathways and biomarkers suggestive of decreased inflammation, decreased neural and epidermal dysregulation, and increased wound healing. Taken together, these results suggest that IL-31 works through multiple pathways affecting inflammatory, neural, and epithelial regulation to contribute to the pathophysiology of PN. By targeting signaling downstream of IL-31RA, nemolizumab represents a promising potential new approach for the clinical management of PN. Future studies involving a larger, fully randomized cohort of patients with PN treated with nemolizumab or placebo are needed to expand our conclusions to nemolizumab response in PN as a whole. Additional studies should also examine the cutaneous effects of nemolizumab therapy, including the modulation of inflammatory, neuronal network, and tissue remodeling pathways, to further characterize the exact mechanisms through which nemolizumab affects PN pathophysiology.

## Notes

Supplement 1. **eFigure 1.** Study Schematic **eFigure 2.** Heat Map of Mean-Centered and Scaled Protein Abundances for the Upregulated and Downregulated Differentially Expressed Proteins **eFigure 3.** Principal Component Analysis Plot of Protein Abundances by Treatment Type and Class **eFigure 4.** Upstream Regulators in the Nemolizumab Group vs the Placebo Group **eTable.** Differentially Expressed Proteins in Nemolizumab Responder vs Placebo Nonresponder Groups

Supplement 2.
Data Sharing Statement
