# Supplementary material for: Modulation of Neuroimmune and Epithelial Dysregulation in Patients With Moderate to Severe Prurigo Nodularis Treated With Nemolizumab
Source: JAMA Dermatol. 2023 Aug 9;159(9):977–85. doi: 10.1001/jamadermatol.2023.2609 (PMC10413221; doi:10.1001/jamadermatol.2023.2609)
Supplement: Supplement 1. — eFigure 1. Study Schematic eFigure 2. Heat Map of Mean-Centered and Scaled Protein Abundances for the Upregulated and Downregulated Differentially Expressed Proteins eFigure 3. Principal Component Analysis Plot of Protein Abundances by Treatment Type and Class eFigure 4. Upstream Regulators in the Nemolizumab Group vs the Placebo Group eTable. Differentially Expressed Proteins in Nemolizumab Responder vs Placebo Nonresponder Groups [file jamadermatol-e232609-s001.pdf]

Supplementary Online Content

Deng J, Liao V, Parthasarathy V, et al. Modulation of neuroimmune and epithelial dysregulation in patients with moderate-to-severe prurigo nodularis treated with nemolizumab. *JAMA Dermatol*. Published online August 9, 2023. doi:10.1001/jamadermatol.2023.2609

eFigure 1. Study Schematic
eFigure 2. Heat Map of Mean-Centered and Scaled Protein Abundances for the Upregulated and Downregulated Differentially Expressed Proteins
eFigure 3. Principal Component Analysis Plot of Protein Abundances by Treatment Type and Class
eFigure 4. Upstream Regulators in the Nemolizumab Group vs the Placebo Group
eTable. Differentially Expressed Proteins in Nemolizumab Responder vs Placebo Nonresponder Groups

This supplementary material has been provided by the authors to give readers additional information about their work.

© 2023 Deng J et al. *JAMA Dermatology*.

eFigure 1. Study Schematic

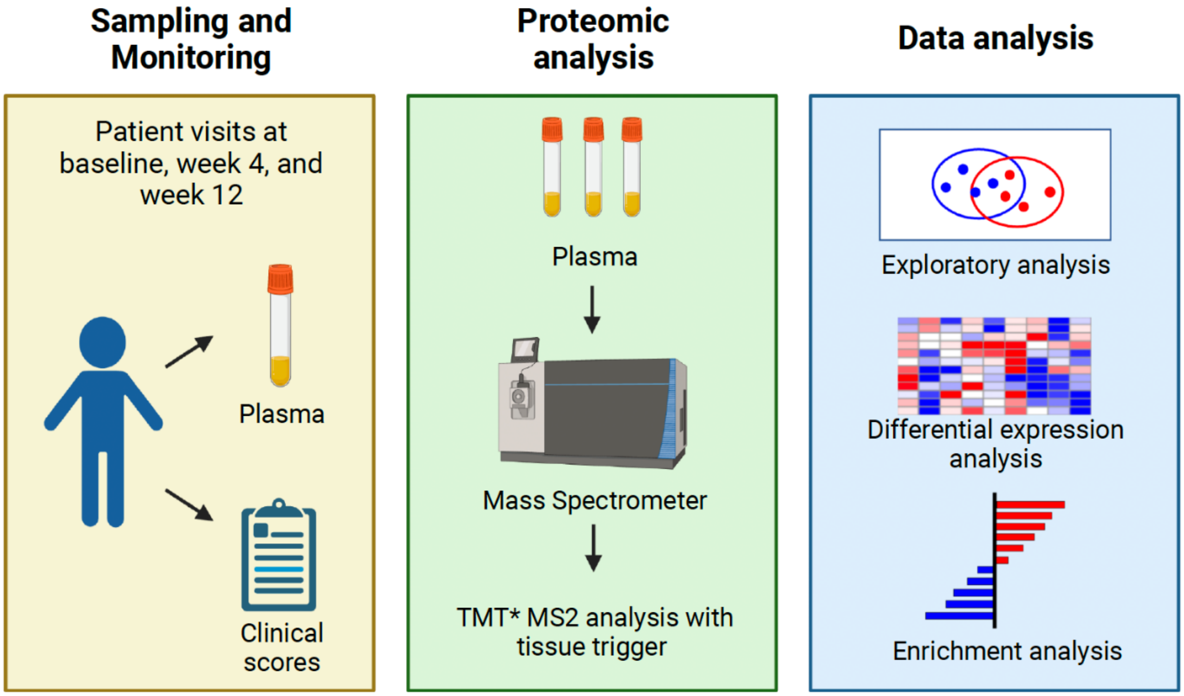

© 2023 Deng J et al. *JAMA Dermatology*.

eFigure 2. Heat Map of Mean-Centered and Scaled Protein Abundances for the Upregulated and Downregulated Differentially Expressed Proteins

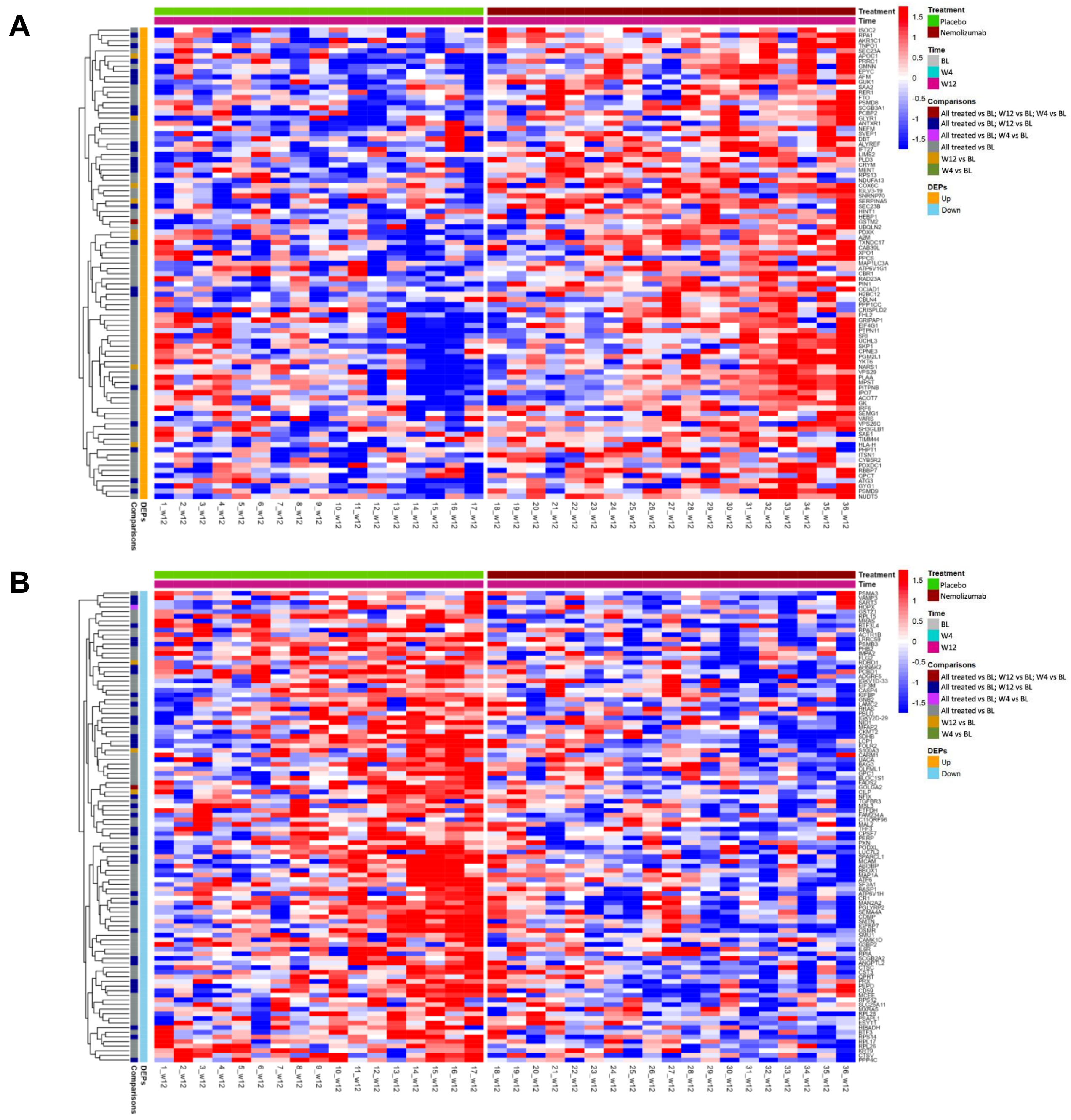

(a) upregulated and (b) downregulated differentially expressed proteins for each individual patient from the nemolizumab responder and placebo non-responder groups at week 12, with correction for the abundances at baseline. Proteins and samples are ordered by hierarchical clustering using Euclidian distances. Red colors represent higher abundance values while blue colors represent lower abundance values.

© 2023 Deng J et al. *JAMA Dermatology*.

eFigure 3. Principal Component Analysis Plot of Protein Abundances by Treatment Type and Class

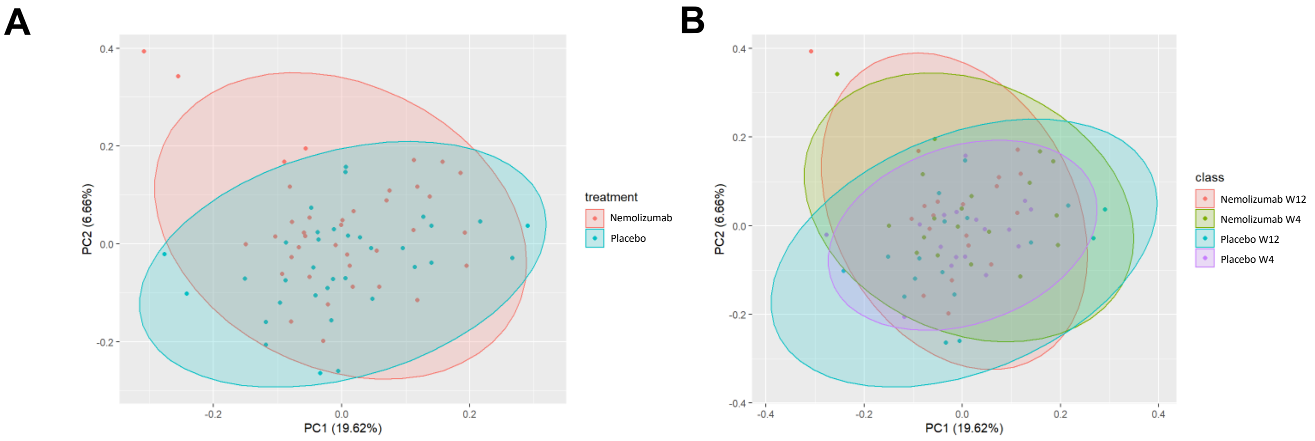

(a) Principal component analysis (PCA) plot of protein abundances by treatment type. (b) PCA plot of protein abundances by class (treatment + time point). Each dot in the PCA plots represents a different sample and each color represents different sample annotations. PC1 and PC2 axis labels indicate the % of variance explained by each one of these components, respectively.

© 2023 Deng J et al. *JAMA Dermatology*.

eFigure 4. Upstream Regulators in the Nemolizumab Group vs the Placebo Group

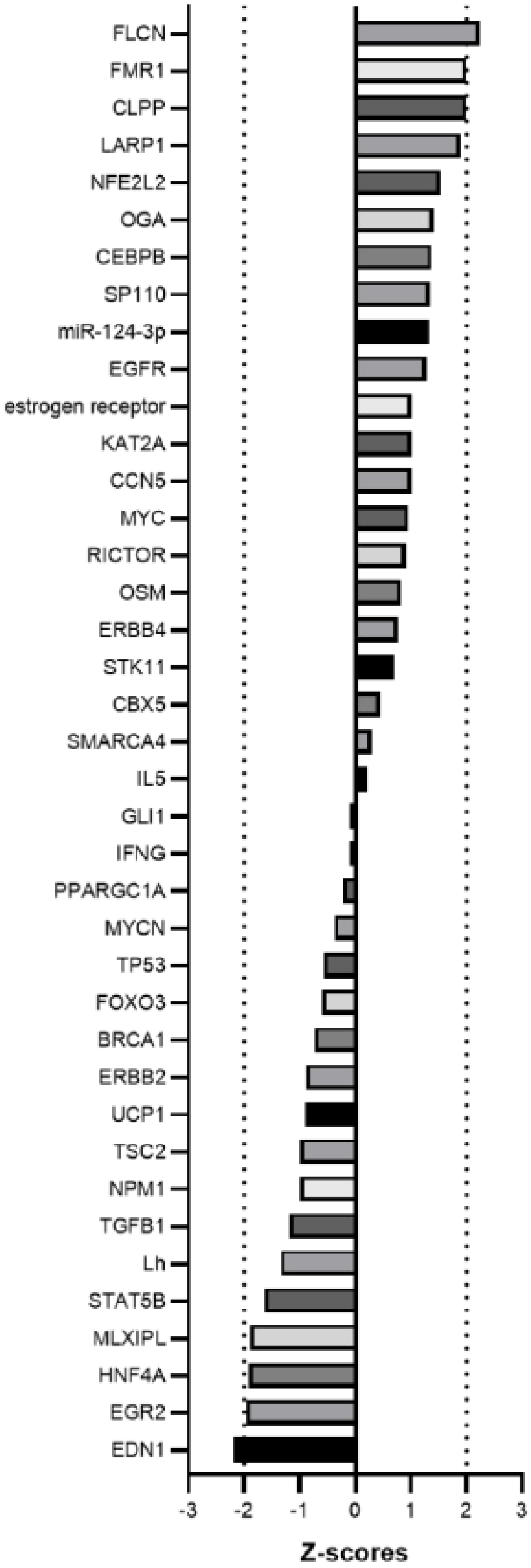

© 2023 Deng J et al. *JAMA Dermatology*.

eTable. Differentially Expressed Proteins in Nemolizumab Responder vs Placebo Nonresponder Groups

Gene Name	Log_2(fold change)	P-value	Adjusted P-value
**IGKV2D-29**	-0.504	0.000047	0.006
**ACOT7**	0.232	0.003	0.049
**PSMD9**	0.399	0.00000038	0.00026
**PODXL**	-0.22	0.00003	0.012
**PDXK**	0.381	0.00065	0.039
**IMPA2**	-0.298	0.00081	0.044
**MRAS**	-0.292	0.002	0.047
**IRF6**	0.27	0.001	0.032
**VPS26C**	0.468	0.000026	0.005
**XPO1**	0.193	0.000037	0.005
**RER1**	0.223	0.001	0.032
**YKT6**	0.291	0.002	0.044
**TIMM44**	0.454	0.0005	0.021
**GSTZ1**	-0.331	0.001	0.032
**NARS1**	0.673	0.00069	0.04
**H2BC12**	0.387	0.0000052	0.003
**CTSV**	-0.363	0.00036	0.03
**CPNE3**	0.174	0.002	0.038
**CILP**	-0.184	0.00052	0.037
**ATP6V1G1**	0.358	0.00094	0.03
**GMNN**	0.416	0.00054	0.037
**BBOX1**	-0.222	0.002	0.045

© 2023 Deng J et al. *JAMA Dermatology*.

**IPO7**	0.366	0.001	0.032
**BAG3**	-0.183	0.001	0.032
**FADS2**	-0.167	0.002	0.048
**A2M**	0.402	0.00094	0.048
**CST3**	-0.132	0.001	0.032
**HRAS**	-0.426	0.00031	0.018
**IGKV1D-33**	-0.458	0.001	0.032
**IGLV3-19**	0.837	0.00084	0.028
**HLA-H**	0.284	0.00065	0.039
**APOC1**	0.688	0.00032	0.031
**SEMG1**	0.461	0.002	0.038
**SERPINA5**	0.436	0.00025	0.027
**NEFM**	0.208	0.00028	0.017
**SNRNP70**	0.123	0.002	0.047
**IL6R**	-0.231	0.001	0.034
**COX6C**	0.284	0.00058	0.039
**SAA2**	0.34	0.003	0.05
**DBT**	0.194	0.001	0.033
**PEPD**	-0.226	0.000031	0.005
**LCP1**	-0.236	5.2E-08	0.000046
**CD59**	-0.292	0.000011	0.003
**FOLR2**	-0.243	0.00016	0.012
**NID1**	-0.145	0.00024	0.016
**UCHL3**	0.235	0.002	0.048

© 2023 Deng J et al. *JAMA Dermatology*.

**CBR1**	0.134	0.00017	0.013
**CKMT2**	-0.5	0.002	0.039
**CR1**	-0.307	0.00051	0.021
**RPL17**	-0.183	0.00012	0.011
**ATF6**	-0.24	0.00008	0.018
**BTF3**	-0.414	0.00016	0.012
**SDHB**	-0.168	0.0000064	0.003
**MPST**	0.249	0.0009	0.044
**RPS12**	-0.191	0.000033	0.005
**PSMA3**	-0.131	0.001	0.037
**VARS**	0.135	0.00045	0.02
**RPA1**	0.278	0.000035	0.005
**GSTM2**	0.27	4.6E-08	0.000046
**PBLD**	-0.274	0.00094	0.03
**SRI**	0.178	0.00028	0.017
**HIBADH**	-0.266	0.000072	0.008
**GK**	0.277	0.00096	0.045
**S100A3**	-0.33	0.00075	0.042
**GPC1**	-0.345	0.002	0.039
**RPA3**	-0.251	0.00067	0.026
**KRT9**	-0.796	0.00075	0.027
**PPP1CC**	0.875	0.00011	0.023
**ACTR1B**	-0.487	0.001	0.034
**MCAM**	-0.145	0.00034	0.019

© 2023 Deng J et al. *JAMA Dermatology*.

**AFM**	0.285	0.000058	0.012
**RPL28**	-0.205	0.00037	0.019
**GYG1**	0.273	0.00038	0.019
**PSMD8**	0.384	0.00035	0.019
**PITPNB**	0.339	0.00002	0.004
**PXN**	-0.458	0.00026	0.017
**RPIA**	-0.24	0.001	0.033
**MAN2A2**	-0.273	0.0000067	0.003
**CASP4**	-0.288	0.00015	0.012
**PSMB3**	-0.236	0.00014	0.018
**COMP**	-0.194	0.00067	0.026
**HINT1**	0.171	0.002	0.044
**CTSC**	-0.13	0.002	0.048
**SMTN**	-0.355	0.00058	0.023
**RAD23A**	0.168	0.002	0.044
**MFAP2**	-0.299	0.00083	0.028
**PPP4C**	-0.344	0.000012	0.003
**RPL26**	-0.632	0.00009	0.009
**RPL15**	-0.194	0.002	0.046
**PCBD1**	-0.248	0.00031	0.018
**RPS14**	-0.416	0.00036	0.031
**RPS13**	0.136	0.00044	0.02
**GNB2**	-0.343	0.00056	0.023
**SKP1**	0.295	0.002	0.047

© 2023 Deng J et al. *JAMA Dermatology*.

**BLOC1S1**	-0.287	0.002	0.039
**MAP1A**	-0.208	0.00077	0.027
**BASP1**	-0.211	0.001	0.032
**SLC25A11**	-0.205	0.00025	0.03
**TGFBR3**	-0.184	0.002	0.046
**EIF4G1**	0.147	0.001	0.036
**AKR1C1**	0.432	0.001	0.033
**PTPN11**	0.255	0.002	0.046
**TFF3**	-0.33	0.00005	0.006
**GOLGA2**	-0.474	3.2E-09	0.000011
**SCGB2A2**	-0.466	0.000014	0.003
**PIN1**	0.265	0.00038	0.019
**LAMC2**	-0.231	0.00069	0.026
**FHL2**	0.271	0.002	0.044
**SPARCL1**	-0.169	0.000066	0.007
**CRYM**	0.303	0.00056	0.023
**NFIX**	-0.328	0.00025	0.016
**SART3**	-0.181	0.00072	0.026
**QPRT**	-0.422	0.0001	0.01
**PCBP2**	0.488	0.00011	0.016
**SEC23A**	0.158	0.002	0.047
**SEC23B**	0.343	0.00013	0.011
**SF3A1**	-0.215	0.00044	0.02
**ITSN1**	0.342	0.0006	0.038

© 2023 Deng J et al. *JAMA Dermatology*.

**VAMP3**	-0.618	5.3E-08	0.000046
**ETFDH**	-0.297	0.00082	0.044
**IGFBP7**	-0.167	0.00061	0.024
**RBBP7**	0.421	0.002	0.044
**QPCT**	0.438	0.00058	0.023
**GUK1**	0.292	0.000047	0.006
**SMU1**	-0.293	0.0004	0.019
**GLYR1**	0.569	0.000072	0.012
**SVEP1**	0.367	0.00012	0.011
**GRIPAP1**	0.613	0.001	0.034
**FLG2**	-0.158	0.00034	0.019
**CYB5R2**	0.207	0.000064	0.007
**PSAPL1**	-0.188	0.002	0.044
**PDXDC1**	0.389	0.00069	0.026
**PGM2L1**	0.392	0.001	0.032
**OLFML1**	-0.171	0.00017	0.013
**EIF3M**	-0.259	0.002	0.044
**LIMS2**	0.423	0.000031	0.005
**ABI3BP**	-0.127	0.002	0.044
**C11ORF96**	-0.446	0.003	0.05
**ALYREF**	0.47	0.00022	0.015
**CARM1**	-0.265	0.002	0.044
**CAMK1D**	-0.443	0.00012	0.011
**PLD3**	0.193	0.00034	0.019

© 2023 Deng J et al. *JAMA Dermatology*.

**AHNAK2**	-0.101	0.00013	0.011
**ADGRF5**	-0.13	0.002	0.039
**MSL3**	-0.291	0.00015	0.012
**CPSF7**	-0.287	0.0007	0.026
**TNPO1**	0.309	0.000023	0.004
**MAL2**	-0.293	0.00099	0.031
**ISOC2**	0.406	0.001	0.034
**LRRC59**	-0.166	0.001	0.034
**KIFBP**	-0.186	0.00039	0.019
**PERP**	-0.375	0.00038	0.019
**BTF3L4**	-0.645	0.0000055	0.016
**PRRC1**	0.48	0.00006	0.012
**PGLYRP2**	-0.104	0.002	0.041
**MCEE**	-0.303	0.00082	0.028
**SCGB3A1**	0.577	0.00034	0.031
**PHB2**	-0.132	0.002	0.044
**EPYC**	0.688	0.000029	0.005
**OSMR**	-0.203	0.00036	0.019
**HOPX**	-0.942	0.00000035	0.00062
**TXNDC17**	0.307	0.0000068	0.003
**ESYT1**	-0.203	0.001	0.032
**MENT**	0.331	0.00017	0.013
**IFT27**	0.368	0.0002	0.014
**PRX**	-0.28	0.00062	0.024

© 2023 Deng J et al. *JAMA Dermatology*.

**UACA**	-0.393	0.003	0.049
**FTO**	0.204	0.00071	0.026
**CRISPLD2**	0.335	0.00045	0.02
**FAM234A**	-0.737	0.000009	0.003
**SEMA4A**	-0.192	0.001	0.032
**MAP1LC3A**	0.304	0.001	0.032
**ANTXR1**	0.35	0.000099	0.01
**CAB39L**	0.366	0.00032	0.03
**PPCS**	0.202	0.002	0.044
**MXRA5**	-0.186	0.001	0.036
**HEBP1**	0.23	0.00082	0.028
**PHPT1**	0.318	0.0000078	0.003
**ATG3**	0.543	0.000031	0.005
**CBLN4**	0.243	0.002	0.044
**OCIAD1**	0.53	0.001	0.033
**NDUFA13**	0.448	0.000019	0.004
**SAE1**	0.176	0.002	0.044
**VPS29**	0.218	0.002	0.047
**UBQLN2**	0.379	0.002	0.039
**ATP6V1H**	-0.353	0.000047	0.012
**NUDT5**	0.171	0.000082	0.009
**ANGPTL2**	-0.413	0.000074	0.012
**G3BP2**	-0.66	0.00048	0.021
**PLAA**	0.331	0.00075	0.027

© 2023 Deng J et al. *JAMA Dermatology*.

**SH3GLB1**	0.35	0.00036	0.019
**LUC7L2**	-0.468	0.0005	0.021
**ROBO1**	-0.256	0.00047	0.036

© 2023 Deng J et al. *JAMA Dermatology*.
