# Supplementary material for: Modulation of Neuroimmune and Epithelial Dysregulation in Patients With Moderate to Severe Prurigo Nodularis Treated With Nemolizumab
Source: JAMA Dermatol. 2023 Aug 9;159(9):977–85. doi: 10.1001/jamadermatol.2023.2609 (PMC10413221; doi:10.1001/jamadermatol.2023.2609)
Supplement: Supplement 2. — Data Sharing Statement [file jamadermatol-e232609-s002.pdf]

Data Sharing Statement

Deng. Modulation of Neuroimmune and Epithelial Dysregulation in Patients With Moderate to Severe Prurigo Nodularis Treated With Nemolizumab. *JAMA Dermatol*. Published August 09, 2023. doi:10.1001/jamadermatol.2023.2609

Data
Data available: No
